# Mixed Neuroendocrine–Non-Neuroendocrine Neoplasm of the Esophagogastric Junction with Enteroblastic Differentiation and Morphologic–Immunophenotypic Discordance: A Case Report with Five-Year Disease-Free Survival

**DOI:** 10.70352/scrj.cr.25-0692

**Published:** 2026-04-07

**Authors:** Junichi Yoshizawa, Seisyu Karasawa, Kenya Nakamura, Kentaro Fukushima, Ataru Nakayama

**Affiliations:** Department of Surgery, Ina Central Hospital, Ina, Nagano, Japan

**Keywords:** mixed neuroendocrine–non-neuroendocrine neoplasm (MiNEN), neuroendocrine carcinoma, esophagogastric junction, enteroblastic differentiation, immunophenotypic discordance, case report

## Abstract

**INTRODUCTION:**

Mixed neuroendocrine–non-neuroendocrine neoplasms (MiNENs) of the esophagogastric junction (EGJ) are extremely rare and are generally associated with aggressive clinical behavior. Enteroblastic (fetal gut-like) differentiation at this anatomical site is uncommon and is regarded as a poor prognostic feature. In addition, discordance between tumor morphology and immunophenotype in such mixed tumors has rarely been documented, and its clinical significance remains unclear. We report an exceptionally rare case of an EGJ MiNEN with enteroblastic differentiation and morphologic–immunophenotypic discordance that achieved long-term disease-free survival after surgery alone.

**CASE PRESENTATION:**

An 81-year-old man presented with progressive dysphagia. Upper gastrointestinal endoscopy revealed a circumferential, ulcerated mass extending from the distal esophagus to the EGJ. Biopsy specimens suggested adenocarcinoma, and preoperative imaging demonstrated a localized EGJ tumor with suspected regional lymph node involvement. The patient underwent transhiatal distal esophagectomy with total gastrectomy, D2 lymphadenectomy, and Roux-en-Y reconstruction. Histopathological examination of the resected specimen demonstrated a MiNEN, with each component accounting for more than 30% of the tumor volume. The glandular component consisted of a well-to-moderately differentiated adenocarcinoma with clear-cell morphology and enteroblastic features, showing positivity for glypican-3 and SALL4 with focal alpha-fetoprotein expression. The neuroendocrine component was a high-grade tumor with a markedly elevated proliferative index. Notably, immunophenotypic overlap was observed between the two components, with expression of neuroendocrine markers in the glandular areas and enteroblastic markers in the neuroendocrine nests. Resection margins were tumor-free. No adjuvant therapy was administered because of the patient’s advanced age and comorbidities. The patient remains alive and disease-free 5 years after surgery.

**CONCLUSIONS:**

This case illustrates an exceptionally rare MiNEN of the EGJ with enteroblastic differentiation and morphologic–immunophenotypic discordance. Despite the presence of aggressive histologic features, long-term disease-free survival was achieved with surgical resection alone. This report highlights the importance of thorough histopathological and immunohistochemical evaluation for accurate diagnosis and suggests that curative surgery alone may provide durable disease control in carefully selected patients with localized disease.

## Abbreviations

AFP
: alpha-fetoprotein
EGJ
: esophagogastric junction
MiNEN
: mixed neuroendocrine–non-neuroendocrine neoplasm
NEC
: neuroendocrine carcinoma
SSTR2A
: somatostain receptor 2A
SUV_max
: maximum standardized uptake value

## INTRODUCTION

MiNENs, formerly classified as mixed adenoneuroendocrine carcinomas (MANECs), are defined as tumors containing both neuroendocrine and non-neuroendocrine epithelial components within the same lesion, according to contemporary classifications.^1)^ Although MiNENs can arise throughout the gastrointestinal tract, involvement of the EGJ is exceptionally rare and has been reported mainly in isolated case reports or small series with heterogeneous histology and clinical outcomes.^2)^ Enteroblastic (fetal gut–like) differentiation, characterized by clear, glycogen-rich cytoplasm and papillotubular architecture with immunophenotypic expression of oncofetal markers (AFP, glypican-3, and SALL4), is associated with frequent lymphovascular invasion, a high incidence of liver metastasis, and poor prognosis compared with conventional adenocarcinoma in gastric cancer.^3)^ Although rare, a small subset of esophageal and EGJ adenocarcinomas has been reported to exhibit fetal gut–like or enteroblastic features, frequently co-expressing claudin-6, glypican-3, and SALL4, indicating that this aggressive oncofetal phenotype can occur at the EGJ.^4)^ However, the coexistence of an enteroblastic-type adenocarcinoma and a high-grade neuroendocrine component within a single EGJ tumor is exceedingly uncommon, and its clinicopathological characteristics and optimal management remain unclear.^2,5)^ In addition, morphologic and immunophenotypic discordance between neuroendocrine and non-neuroendocrine components in MiNENs has rarely been described, despite its potential implications for tumor pathogenesis and diagnostic accuracy.^6)^ Against this background, we report an exceptionally rare case of an EGJ MiNEN composed of an adenocarcinoma with enteroblastic differentiation and a high-grade NEC, which achieved more than 5 years of disease-free survival after curative surgery alone. This case highlights important diagnostic considerations and provides insight into the biological heterogeneity of MiNENs arising at the EGJ.

## CASE PRESENTATION

An 81-year-old Japanese man with a history of hypertension, type 2 diabetes mellitus, and interstitial pneumonia presented with a 1-month history of progressive dysphagia to solid foods. The physical examination was unremarkable. Laboratory studies showed hemoglobin (Hb) 14.4 g/dL and HbA1c 6.7%, with other routine blood chemistry values within normal limits. Tumor markers included a mildly elevated carcinoembryonic antigen level of 7.4 ng/mL (normal <5.0) and a normal carbohydrate antigen 19-9 level. Serum AFP level was not assessed preoperatively.

**Fig. 1:**
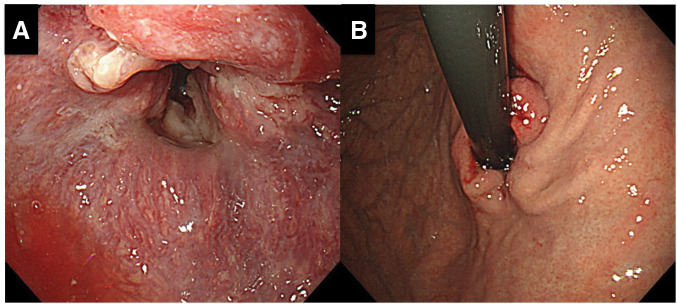
Endoscopic views of the EGJ tumor. (**A**) A close-up endoscopic image of the distal esophagus and EGJ shows a nearly circumferential, ulcerated, ridged mass protruding into the lumen with irregular, friable mucosa. (**B**) An oblique endoscopic view demonstrates the tumor spanning the gastroesophageal junction; the ulcerated, nodular lesion causes luminal narrowing. These findings are consistent with a large ulceroinfiltrative carcinoma involving the distal esophagus/EGJ. EGJ, esophagogastric junction

**Fig. 2:**
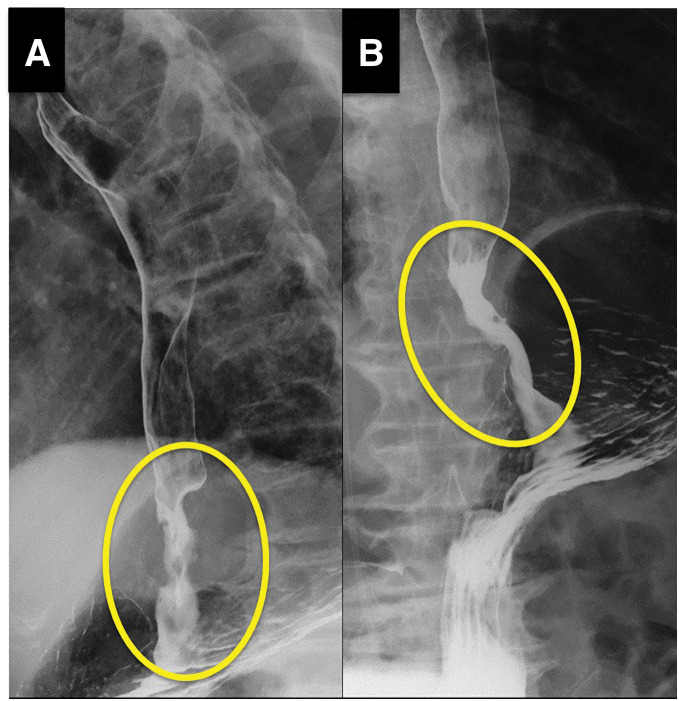
Upper gastrointestinal barium swallow series. The yellow circle indicates the stenotic lesion at the EGJ. (**A**) Lateral projection shows an irregular, eccentric stricture of the distal esophagus/EGJ with abrupt luminal narrowing and an uneven mucosal contour. (**B**) Anteroposterior view confirms the stenotic segment at the gastroesophageal junction, which has an irregular, serrated outline. These contrast images highlight the asymmetric, ragged narrowing caused by the tumor. EGJ, esophagogastric junction

Esophagogastroduodenoscopy revealed an almost circumferential, ulcerated mass extending from the distal esophagus across the EGJ (approximately 33–40 cm from the incisors), causing significant luminal narrowing (**Fig. 1A** and **1B**). Biopsy of the lesion confirmed a well-to-moderately differentiated tubular adenocarcinoma. An upper gastrointestinal barium swallow demonstrated an irregular, eccentric stricture involving the distal esophagus and EGJ, with an abrupt transition and an uneven mucosal contour (**Fig. 2A** and **2B**).

**Fig. 3:**
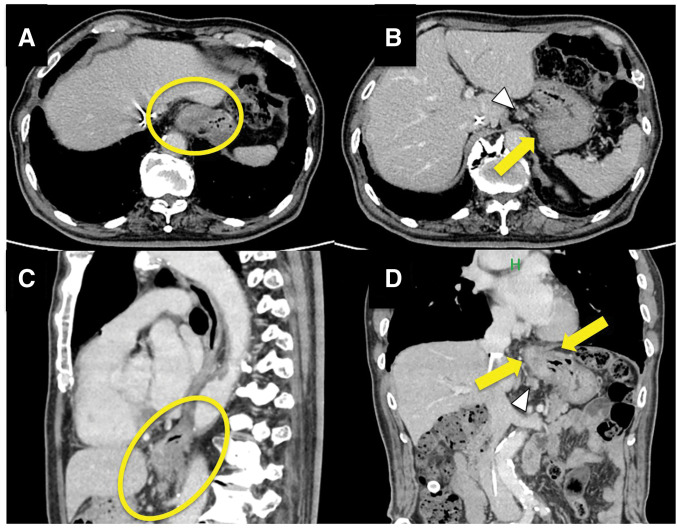
Contrast-enhanced CT of the chest and abdomen. The yellow circles in panels **A** and **C** indicate the primary tumor, and the yellow arrows in panels **B** and **D** indicate the enlarged perigastric lymph nodes. (**A**, **B**) Axial images reveal concentric wall thickening of the lower thoracic esophagus (**A**) and an irregular enhancing mass at the gastric cardia (**B**). The enlarged perigastric lymph node is indicated by an arrowhead in panel (**B**). (**C**) Sagittal reconstruction shows longitudinal tumor extension from the distal esophagus through the EGJ. (**D**) Coronal reconstruction demonstrates continuous tumor infiltration across the EGJ and enlarged perigastric lymph nodes (arrowheads), suggestive of regional nodal metastasis. EGJ, esophagogastric junction

**Fig. 4:**
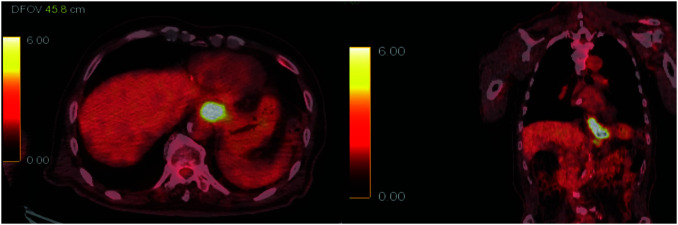
Fluorine-18 FDG PET/CT fusion image (axial slice). Intense FDG uptake is seen in the distal esophageal/EGJ tumor (SUV ~12.6). EGJ, esophagogastric junction; FDG, fluorodeoxyglucose; SUV, standardized uptake value

Contrast-enhanced CT of the chest and abdomen revealed concentric thickening of the lower thoracic esophagus extending into the gastric cardia, as well as an enlarged lymph node along the lesser curvature of the stomach that was suspicious for regional metastasis (**Fig. 3A**–**3D**). No other enlarged lymph nodes were detected, and there was no evidence of distant metastasis to the lungs or liver. PET-CT demonstrated intense fluorodeoxyglucose uptake in the lower esophagus/EGJ region (SUV_max ~12.6) and moderate uptake in the lesser curvature lymph node (SUV_max ~4.3) (**Fig. 4**). These findings were consistent with a locally advanced Siewert type II EGJ adenocarcinoma. The clinical stage was cT3N1M0, corresponding to Stage IIIB disease according to the 8th edition of the Union for International Cancer Control TNM classification (TNM, UICC 8th edition).

Given the patient’s advanced age, underlying interstitial pneumonia, and personal preference, an upfront surgical resection was planned without neoadjuvant therapy. He subsequently underwent a transhiatal distal esophagectomy with total gastrectomy, en bloc D2 lymphadenectomy, and Roux-en-Y esophagojejunostomy reconstruction. The operation was completed successfully with no intraoperative complications.

**Fig. 5:**
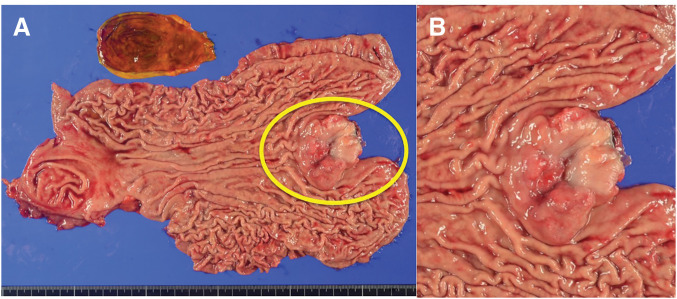
Gross pathology of the resected esophagogastric specimen. The yellow circle in panel **A** indicates the tumor. (**A**) Whole-mount view of the opened distal esophagus and proximal stomach showing a 4.5 × 3.2 cm ulceroinfiltrative tumor spanning the esophagogastric junction. The lesion has an irregular, fungating border with central ulceration. (**B**) Close-up of the tumor (same orientation) shows a raised, nodular mass with an ulcerated surface and irregular edges. A ruler (bottom) provides scale (10 mm increments).

On gross examination of the resected specimen, an ulceroinfiltrative tumor measuring 4.5 × 3.2 cm was identified at the EGJ, extending from the distal esophagus into the gastric cardia (a Borrmann type 3 lesion) (**Fig. 5**). The tumor had a nodular, ulcerated surface with irregular invasive margins. Histologically, the neoplasm consisted of two distinct yet intermingled components: an adenocarcinoma with enteroblastic differentiation and a high-grade NEC.

**Fig. 6:**
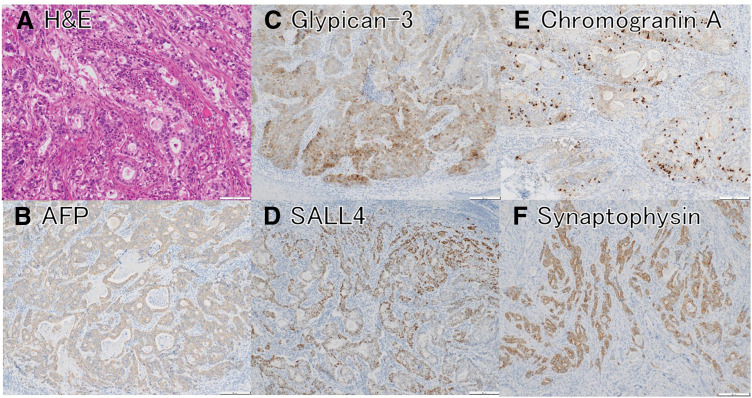
Histology and immunohistochemistry of the adenocarcinoma (enteroblastic) component. (**A**) H&E stain shows irregular papillotubular tumor glands with cells containing abundant, clear, glycogen-rich cytoplasm (enteroblastic differentiation). (**B**) AFP immunostain shows focal weak cytoplasmic positivity (brown) in a subset of the glandular tumor cells, consistent with oncofetal differentiation. (**C**) Glypican-3 immunostain shows diffuse cytoplasmic positivity (brown) in the tumor cells, confirming oncofetal differentiation. (**D**) SALL4 immunostain shows strong nuclear labeling (brown) of the tumor cell nuclei, consistent with a fetal gut–like phenotype. (**E**) Chromogranin A immunostain shows granular cytoplasmic positivity (brown) in some tumor cells (patchy/focal), indicating focal neuroendocrine differentiation. (**F**) Synaptophysin immunostain shows diffuse strong cytoplasmic positivity (brown) in the tumor nests, confirming neuroendocrine differentiation. AFP, alpha-fetoprotein; H&E, hematoxylin and eosin

The adenocarcinoma component predominated on the esophageal side and was a well-to-moderately differentiated glandular tumor with an irregular papillotubular architecture. Many of the tumor cells in this component had abundant clear, glycogen-rich cytoplasm reminiscent of fetal gastrointestinal epithelium. Immunohistochemistry confirmed enteroblastic (fetal gut–like) differentiation, with diffuse cytoplasmic glypican-3 and strong nuclear SALL4 expression, and focal weak positivity for AFP. Notably, a minor subset of the adenocarcinoma cells also exhibited focal positivity for the neuroendocrine markers chromogranin A and synaptophysin (**Fig. 6A**–**6F**).

**Fig. 7:**
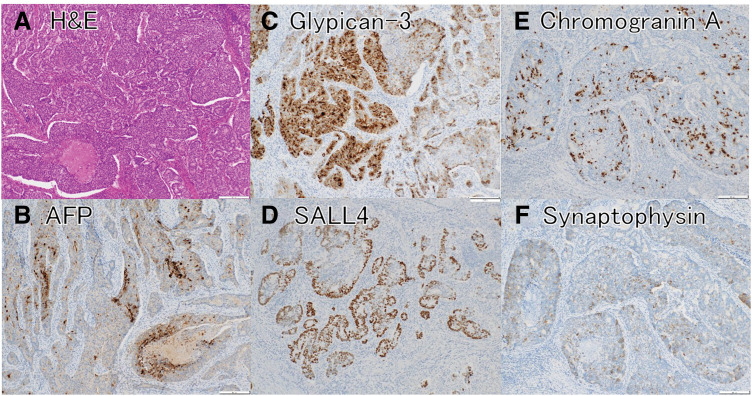
Histology and immunohistochemistry of the high-grade NEC component. (**A**) H&E stain shows solid sheets and nests of atypical cells with organoid (rosette-like) structures and high-grade nuclei. (**B**) AFP immunostain shows focal cytoplasmic positivity (brown) in a subset of tumor cells, indicating oncofetal marker expression within the NEC component. (**C**) Glypican-3 immunostain shows patchy cytoplasmic positivity (brown) in the tumor cells, reflecting partial oncofetal differentiation. (**D**) SALL4 immunostain shows scattered tumor cell nuclei with positive staining (brown), indicating oncofetal marker expression even in the neuroendocrine component. (**E**) Chromogranin A immunostain shows granular cytoplasmic positivity (brown) in some tumor cells (patchy/focal), consistent with neuroendocrine differentiation. (**F**) Synaptophysin immunostain shows diffuse strong cytoplasmic positivity (brown) in the tumor nests, further confirming neuroendocrine differentiation. AFP, alpha-fetoprotein; H&E, hematoxylin and eosin; NEC, neuroendocrine carcinoma

**Fig. 8:**
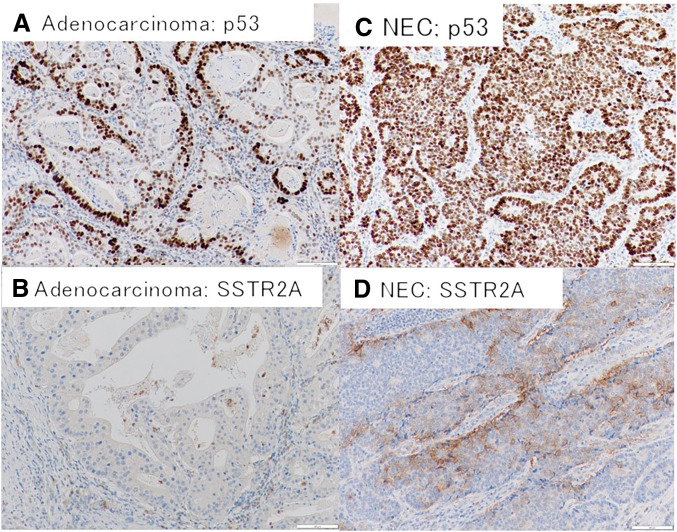
Additional immunohistochemical staining supporting classification of the neuroendocrine component as NEC. (**A**) p53 immunostain in the adenocarcinoma (enteroblastic) component shows nuclear immunoreactivity in tumor cells. (**B**) SSTR2A immunostain in the adenocarcinoma component is negative. (**C**) p53 immunostain in the neuroendocrine component shows diffuse strong nuclear positivity. (**D**) SSTR2A immunostain shows partial membranous positivity in the neuroendocrine component. Scale bars = 100 μm. NEC, neuroendocrine carcinoma; SSTR2A, somatostatin receptor 2A

The high-grade NEC component was mainly located in the gastric side of the tumor, forming solid sheets and nests of poorly differentiated cells with a high nuclear-to-cytoplasmic ratio, rosette-like organoid structures, and foci of necrosis. The Ki-67 proliferation index in this neuroendocrine component was approximately 80%–90%, reflecting its highly proliferative nature. Immunohistochemical staining demonstrated diffuse strong synaptophysin positivity and patchy chromogranin A positivity, confirming neuroendocrine differentiation. This component also showed focal expression of the oncofetal markers glypican-3, SALL4, and AFP (**Fig. 7A**–**7F**). Additional immunohistochemical staining demonstrated diffuse p53 nuclear positivity in the neuroendocrine component and partial positivity for SSTR2A. SSTR2A was negative in the adenocarcinoma component (**Fig. 8**).

Each tumor component accounted for more than 30% of the overall tumor volume, fulfilling the World Health Organization (WHO) criteria for a MiNEN. Transitional areas between the glandular and neuroendocrine elements were observed microscopically, and lymphovascular invasion was present. All resection margins were free of tumor (R0 resection). The final pathological stage was pT3N1M0, corresponding to Stage IIIB disease (TNM, UICC 8th edition). Histological examination of the metastatic lymph node revealed that the NEC component was predominant, with a possible minor admixture of adenocarcinoma elements.

The postoperative course was uneventful, and the patient was discharged on POD 13. Given the patient’s history of interstitial pneumonia, no adjuvant chemotherapy was administered. At 5 years after surgery, he remains alive and disease-free with no evidence of recurrence.

## DISCUSSION

MiNENs of the EGJ are extremely rare; the literature consists of only sporadic case reports and small series with heterogeneous histology and management approaches.^2)^ The clinicopathological features of previously reported EGJ MiNEN cases are summarized in **Table 1**. Within this already uncommon group, our case is notable for its enteroblastic (fetal gut–like) differentiation in the adenocarcinoma component—an oncofetal phenotype (AFP, GPC3, SALL4 expression) well-documented in gastric cancers but only rarely in esophageal/EGJ tumors.^3,4)^ The coexistence of this enteroblastic-type adenocarcinoma with a high-grade NEC in one lesion is exceptionally uncommon. Achieving a >5-year disease-free survival with surgery alone in such a case is likewise remarkable, given the generally aggressive behavior of high-grade gastroenteropancreatic neuroendocrine neoplasms and mixed tumors.^7)^ NET G3 and NEC are distinct entities despite sharing a Ki-67 threshold of >20%, and distinction relies primarily on morphologic differentiation in the current WHO framework.^8)^ In our case, the poorly differentiated morphology with a markedly elevated Ki-67 index, together with diffuse p53 positivity, supported a diagnosis of NEC rather than NET G3.^9)^ SSTR2A showed only partial positivity, which does not exclude NEC and may be variable in high-grade neoplasms. Notably, p53 immunoreactivity was also observed in the adenocarcinoma component, which may be compatible with a shared clonal background and divergent differentiation within a single neoplasm.^8)^

**Table 1:** Summary of previously reported cases of EGJ MiNEN/MANEC

Case (author, year)	Age	Sex	Tumor features (components/Ki-67)	Stage	Treatment	Outcome
Veits et al. (2013)^10)^	68	Male	Barrett-related adenocarcinoma overlying poorly differentiated NEC	pT1b	ESD	Residual deep NEC; short follow-up
Juanmartiñena et al. (2017)^21)^	57	Male	Poorly differentiated adenocarcinoma + small-cell NEC	pT3N3M0	Ivor–Lewis esophagectomy adjuvant chemoradiotherapy	Alive, disease-free at 8 months
Ambesh et al. (2017)^20)^	67	Male	~60% NEC + ~40% adenocarcinoma	NA	Supportive care	Died; very poor prognosis noted
Yamamoto et al. (2018)^13)^	81	Male	Well-differentiated adenocarcinoma + NEC (Ki-67 >90%)	pT3N0M0	Thoracoscopic esophagectomy	Liver mets at 6 months; died at 8 months
Alamin et al. (2024)^19)^	52	Male	MiNEN at EGJ	T1bN0	ESD + carboplatin/paclitaxel + radiotherapy	Disease-free at 1 year
Takenaka et al. (2024)^2)^	81	Male	EGJ MiNEN (mixed)	Stage IIIA	R0 resection adjuvant S-1 (1 year)	RFS 5.5 years; late relapse at Year 6
Present case (2025)	81	Male	Enteroblastic-type adenocarcinoma + NEC (Ki-67 ~80%–90%); both >30%	pT3N1M0	Surgery alone (R0)	Disease-free at 5 years

EGJ, esophagogastric junction; ESD, endoscopic submucosal dissection; mets, metastasis; MANEC, mixed adenoneuroendocrine carcinoma; MiNEN, mixed neuroendocrine–non-neuroendocrine neoplasm; NA, not applicable; NEC, neuroendocrine carcinoma; RFS, relapse-free survival

In the current WHO classification, any tumor containing both a neuroendocrine and a non-neuroendocrine epithelial component is termed a MiNEN, a definition that applies across different histologic combinations.^1)^ One persistent diagnostic challenge is the under-recognition of MiNEN on biopsy. Superficial endoscopic samples often capture only the more differentiated component (e.g., glandular or squamous epithelium), leading to a misdiagnosis of a “pure” carcinoma.

In several EGJ cases, the neuroendocrine component became evident only upon examination of the resected specimen.^2,10)^ These diagnostic pitfalls underscore the importance of generous sampling and a low threshold for immunohistochemical stains when an EGJ tumor biopsy shows solid growth, marked atypia, or a high proliferative index. In particular, applying both neuroendocrine markers (chromogranin, synaptophysin, CD56) and oncofetal markers (AFP, GPC3, SALL4) can unmask a mixed phenotype and guide the surgical plan.^2,11)^

Histologically, our tumor showed an intimate admixture of the two components with focal transition zones. The adenocarcinoma component exhibited classic enteroblastic features—clear, glycogen-rich cytoplasm and papillotubular (fetal gut–like) architecture—with strong GPC3 and SALL4 expression (and focal AFP positivity), mirroring the phenotype of enteroblastic or AFP-producing gastric adenocarcinomas.^3)^ In gastric cancer, this oncofetal differentiation is associated with frequent lymphovascular invasion, a propensity for liver metastasis, and worse survival compared to conventional adenocarcinoma.^3)^ Notably, although uncommon, an oncofetal phenotype can occur in esophageal/EGJ adenocarcinomas.^4)^

In most mixed adenocarcinoma–NEC tumors, oncofetal markers are largely confined to the glandular component while neuroendocrine markers are expressed in the NEC component.^12)^ Our case largely followed this pattern: the neuroendocrine compartment had an extremely high Ki-67 index (~85%) and only patchy chromogranin/synaptophysin positivity, consistent with the heterogeneous, poorly differentiated nature of high-grade NEC.^7,13)^

Immunophenotypic overlap was observed between the morphologically distinct glandular and neuroendocrine components. Neuroendocrine markers were expressed within the glandular areas, whereas oncofetal markers were detected within the neuroendocrine component, demonstrating reciprocal marker expression across histologic elements. This finding indicates a degree of biphenotypic (amphicrine) differentiation, suggesting that both components arose from a common clone with lineage plasticity.^6)^ Accordingly, even limited immunophenotypic overlap should be interpreted as evidence of a single biphenotypic neoplasm rather than two colliding tumors. In practice, management is directed by the most aggressive histologic component, while still recognizing the enteroblastic element for accurate classification and follow-up (e.g., monitoring serum AFP if the tumor is producing oncofetal proteins).^1,6)^

Distinguishing a true MiNEN from an adenocarcinoma with mere focal neuroendocrine cell clusters has important implications. The WHO emphasizes a quantitative criterion (each component comprising ≥30% of the tumor) to define MiNEN, since scattered neuroendocrine cells in an adenocarcinoma (or vice versa) do not qualify.^1)^ In our case, both components clearly exceeded this threshold and showed obvious transition areas, supporting a genuine composite tumor rather than separate collision tumors. Additionally, the enteroblastic pattern must be differentiated from other oncofetal-rich variants like hepatoid or yolk sac tumor-like adenocarcinoma. All of these variants can express AFP, GPC3, and SALL4, but differences in overall architecture, clinical context, and (in our case) the presence of a concurrent neuroendocrine component favored the interpretation of an enteroblastic adenocarcinoma.^14)^

Prognostically, both the high-grade NEC and the enteroblastic adenocarcinoma components are associated with aggressive behavior and poor outcomes.^3,7)^ Even after an R0 resection, early hepatic relapse and shortened survival have been frequently reported in both mixed tumors and AFP-producing (enteroblastic/hepatoid) variants, and overall prognosis is generally worse than that of a typical EGJ adenocarcinoma. In many cases, the neuroendocrine component ultimately drives the clinical course. Complete resection, however, can yield prolonged remission in selected patients with truly localized disease.^2)^ Our patient remains alive and disease-free 5 years after an R0 resection with no adjuvant therapy. This outcome contrasts with some prior EGJ MiNEN cases that experienced early postoperative recurrence (including hepatic metastases within a year in one report),^13)^ underscoring the heterogeneity of this entity.

There are no EGJ-specific clinical trials to guide therapy, so management is extrapolated from related contexts. For fit patients, radical surgical resection with adequate lymphadenectomy remains the cornerstone of treatment when the tumor is resectable.^1,11)^ Given the high risk of occult systemic spread driven by the neuroendocrine component, many centers advocate adding platinum-based combination chemotherapy (etoposide–cisplatin or irinotecan–cisplatin) in the neoadjuvant or adjuvant setting, by analogy to treatment of high-grade gastrointestinal NECs. This approach is supported by a randomized phase III trial demonstrating comparable efficacy of etoposide–cisplatin versus irinotecan–cisplatin regimens.^15)^ Retrospective analyses have similarly shown the benefit of systemic chemotherapy in advanced disease.^16)^

In the limited literature on EGJ MiNEN, multimodal treatment strategies are common.^17)^ For example, Takenaka et al. reported 5.5 years of recurrence-free survival after esophagectomy followed by one year of adjuvant S-1 (oral fluoropyrimidine), with a late recurrence in the sixth year.^2)^ There are also reports of curative endoscopic resection for superficially invasive lesions and of effective definitive chemoradiotherapy for unresectable cases.^17–21)^

In our case, durable remission with surgery alone highlights the need to individualize therapy. It suggests that in an elderly patient with a completely resected, localized MiNEN and significant comorbidities, a surveillance-only approach can be reasonable after thorough multidisciplinary discussion—whereas in more advanced or high-risk cases, multimodal therapy should be strongly considered. Indeed, no standard adjuvant regimen exists for MiNEN due to its rarity; decisions must be individualized and guided by the most aggressive component of the tumor.^6)^ From a surgical decision-making perspective, this case underscores that achievement of complete (R0) resection is a critical determinant of outcome in EGJ MiNEN. Even in tumors with high-grade neuroendocrine features, durable disease control may be achievable when the disease is truly localized and radical resection is accomplished. Importantly, precise histopathologic assessment—including evaluation of the dominant component and the composition of metastatic lymph nodes—may have implications for postoperative therapeutic planning. In our case, lymph node metastasis was predominantly composed of the NEC component, with possible minor adenocarcinoma admixture, suggesting that metastatic behavior was primarily driven by the NEC. In elderly patients with substantial comorbidities, individualized risk–benefit assessment may support surveillance alone after curative surgery, whereas younger or medically fit patients may warrant consideration of multimodal therapy.

For the diagnostic team, maintaining a high index of suspicion is critical when evaluating EGJ tumor biopsies with mixed or unusual features (e.g., glandular and solid areas, clear-cell change, or a very high Ki-67 index). Performing a dual immunohistochemical panel targeting both neuroendocrine and oncofetal markers on such biopsies can prevent misclassification and ensure proper surgical planning.^2,11)^ For oncologists and surgeons, factors such as the patient’s overall condition, tumor stage (especially nodal involvement), margin status, lymphovascular invasion, and proliferation index should all be weighed in formulating treatment—recognizing that the most poorly differentiated component should guide systemic therapy choices.^1,6)^ Postoperatively, follow-up can be guided by protocols for conventional esophagogastric adenocarcinoma and high-grade NEC, with the addition of serum AFP monitoring if the tumor had oncofetal (AFP-producing) features.

Finally, we acknowledge the limitations of this report. It details a single case and lacks molecular analysis to confirm clonality or identify actionable mutations. Larger series and collaborative studies are needed to determine optimal management strategies (surgery alone vs. multimodal therapy) for EGJ MiNEN and to discover biomarkers that can refine adjuvant treatment decisions. Nonetheless, our case adds to the scant literature by demonstrating that an EGJ MiNEN with enteroblastic differentiation achieved long-term remission with surgery alone. This underscores that meticulous pathology work-up, complete resection, and judicious, patient-specific therapy planning can together yield durable disease control in select patients. Although our patient has remained disease-free for 5 years without adjuvant therapy, this milestone does not necessarily indicate a definitive cure. As reported by Takenaka et al.,^2)^ a similar EGJ MiNEN case experienced late recurrence in the sixth postoperative year despite adjuvant S-1 chemotherapy. Given the potential for late relapse in MiNEN, long-term surveillance beyond 5 years is warranted.

## CONCLUSIONS

EGJ MiNENs with enteroblastic (oncofetal) differentiation are exceptionally rare and often histologically complex. This case illustrates the coexistence of distinctly differentiated glandular and neuroendocrine tumor components, along with a degree of immunophenotypic overlap—highlighting the concept of lineage plasticity within a single neoplasm. An accurate diagnosis required generous tissue sampling and a combined immunohistochemical workup targeting both neuroendocrine and oncofetal markers. This morphologic–immunophenotypic discordance may have important implications for limited biopsy specimens, as sampling of only one component could potentially lead to misclassification and inappropriate therapeutic decision-making. Despite the typically poor prognosis associated with high-grade neuroendocrine and enteroblastic histology, long-term disease-free survival can be achieved with surgery alone in select patients who have completely resected, localized disease. Greater awareness of morphologic and immunophenotypic discordance in these tumors—and continued investigative efforts, including molecular studies—will be essential to refine classification and inform future therapeutic strategies for this challenging entity.

## DECLARATIONS

### Availability of data and materials

All data generated or analyzed during this study are available from the corresponding author on reasonable request.
